# Extract from the Weekly Return of the Sick and Wounded of His Majesty's Ship, Atlas. Dated off Brest, November 20, 1799

**Published:** 1800-03

**Authors:** D. Whyte

**Affiliations:** Atlas, Torbay


### To the Editors of the Medical and Phyſical Journal.

GENTLEMEN,

YOU did me the honour, in a former Number, to inſert my letter to the DUKE of YORK on the treatment of Dyſentery. I now incloſe a few additional reflections on the ſame ſubject, extracted extracted from my report of last week, to the Commissioners for Sick and Wounded Seamen. By inserting them also, you wil oblige,

GENTLEMEN,

Your most obedient servant,

*Atlas, Torbay, Nov.* 30, 1799. D. WHYTE.

---

*Extract from the Weekly Return of the Sick and Wounded of His Majesty's Ship, Atlas. Dated off Brest, November* 20, 1799.

It has been universally remarked, that dysenteries are more frequent in Autumn than any other season; and it may be asked, to what cause is this prevalence to be ascribed? I would answer, that the intestines participate in the general debility induced by the preceding summer heat; and that, as the sun recedes from our hemisphere, we are many of us too neglectful of increasing, with the decrease of temperature, the quantity and consistence of our cloathing. In most seamen, to the predisposing debility occasioned by heat, is superadded and combined the predisposition arising from scurvy. To excite disease in persons so circumstanced, the slightest exposure to cold and moisture is alone necessary.

In a former return, I noticed to you my new method of successfully managing this otherwise dreadful and almost irresistible disease. My experience during the last fortnight, not only confirms the deductions I at that time made, but the circumstances of some of the cases have led to still farther practical improvements; the chief of which is, in the first place, and previous to the application of the flannel rollers, to invest the whole of the abdominal and lumbar regions with a large and closely applied adhesive plaster. Where the disease has been of several days standing, similar plasters applied over the glutœi muscles, and some way down the thighs, are singularly efficacious; enemas of flour and water, or of starch, or of arrow root, are also in such cases useful.

In an irregular patient, I found that wine, ardent spirits, and high seasoned food, exacerbated every symptom; and had he not at last been prevailed upon to leave off those pernicious stimuli, his life would in all probability have been paid as the forfeit of his folly. The disease was also exacerbated by every fluid of a high temperature, while the same fluid cooled to, or below the temperature of 96° could be drank with comparative impunity. By the abstraction of sixty ounces of blood, this this patient was put out of danger; and fo eafily was the fupervening debility removed by proper regimen, that in five days he was able to return to his ufual occupations. Had he been regular from the beginning, the cure (as he was of a weak conftitution, and eafily affected by remedies, as well as exciting caufes) might have been completed in a few hours without any, or, at leaft, with a very moderate venæfection.

Dilution I do not now find neceffary; I am even inclined to believe that it is always more or lefs hurtful.

As articles of diet, animal and vegetable mucilages ought on every principle of reafon and humanity to be alone employed. Beef tea, chicken and mutton broth, boiled to the confiftence of a jelly, are of all others moft palatable and proper. In my prefent humble practice, however, the generality of my patients are under the neceffity of refting contented with portable foup, fago, and flour and water.

Much of what I have here faid is referable only to neglected cafes. Where the patient makes his application on the day of attack, avoids further expofure, and leaves off diffufive and other ftrong ftimuli, an adhefive plafter applied to the abdominal furface, over that a few folds of flannel roller, with a fingle venæfection, moderate or copious according to circumftances, will in moft cafes be fully fufficient for the removal of every fymptom of dyfentery.

I have in the firft place to obferve, that large quantities of acid, or alkali, or of any of the neutral falts formed by their combination, will, after paffing the pylorus, by their direct ftimulus, excite frequent dejections, accompanied, if long continued, with intenfe tormina, tenefmus, and every other dyfenteric fymptom; but I have to obferve, in the fecond place, that the generality of dyfenteries owe not their origin to the direct ftimulus of fuch caufes; and thirdly, that where they do, as fuch fubftances neceffarily operate their own difcharge, alkalies are not the moft proper remedies when the difeafe is excited by acids; nor acids when it is caufed by alkalies. Befides, as I have already mentioned, the acid and alkali may prove purgative, even when combined.

That the tranfatlantic fpecific for dyfentery has a tendency to excite it, I have had frequent opportunities of obferving.

For the removal of acidity of the ftomach, to which feveral of the gentlemen of this fhip are extremely fubject, I have long had daily occafion to exhibit aqua kali; and I have found, that in certain conditions of the weather, and of the patient's irritability, it has occafioned griping and diarrhœa, which, if not timely checked by very different remedies from thofe propofed by the very eloquent Doctor Mitchill, might have

 degenerated degenerated into dyſentery ſtrictly ſo called. In farther confirmation, I may add, that the irregular patient above alluded to had for upwards of a year been, for the removal of arthritic prediſpoſition, in the habit of taking half a drachm of kali ppt. the dyſenteric ſpecific of Dr. Mitchill, twice a day. He had more than once, when in cold and moiſt weather lightly cloathed, perceived its inſtant purgative effect; but, on falling ſick, the fruit of dear-bought experience was of no avail; his faculties became impaired, and he was no longer capable of regulating his conduct by the rules of ſcience. Unaware of the danger, he was fooliſh enough to take, on the evening of attack, his cuſtomary alkaline doſe, and alſo twice to repeat it on the following day. Each time, as *a priori* might have been expected, it increaſed both purging and tormina.

Such are the arguments I have derived from experience, in oppoſition to the doctrine of the ingenious Dr. Mitchill: I am alſo prepared to combat them in theory; but this I muſt defer till I can enjoy a leiſure and retirement more propitious to literary purſuits than my preſent confined and laborious ſituation will admit.

