# Errata

**Published:** 1800-04

**Authors:** 


### ERRATA.

P. 233, l. 25, previous to the words "I have in the first place to observe," we are requested by the Author of that paper, to insert the following preamble to the paragraph:

"With respect to the transatlantic doctrine of this disease, being always, or "for the most part, directly excited by the application of nitrous, azotous, or ac- "cording to the *Mitchillian* nomenclature, *septous acid gas,* and to the assertion of "its being cured by alkalies," I have, &c.

